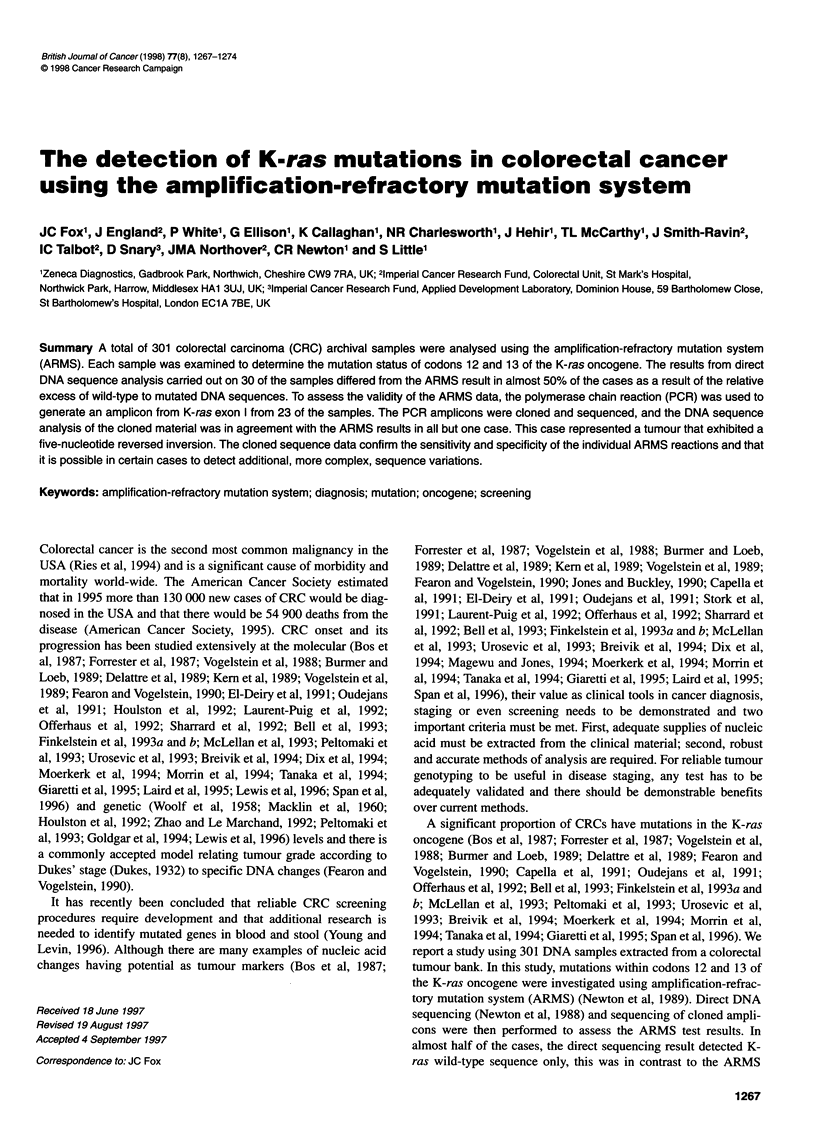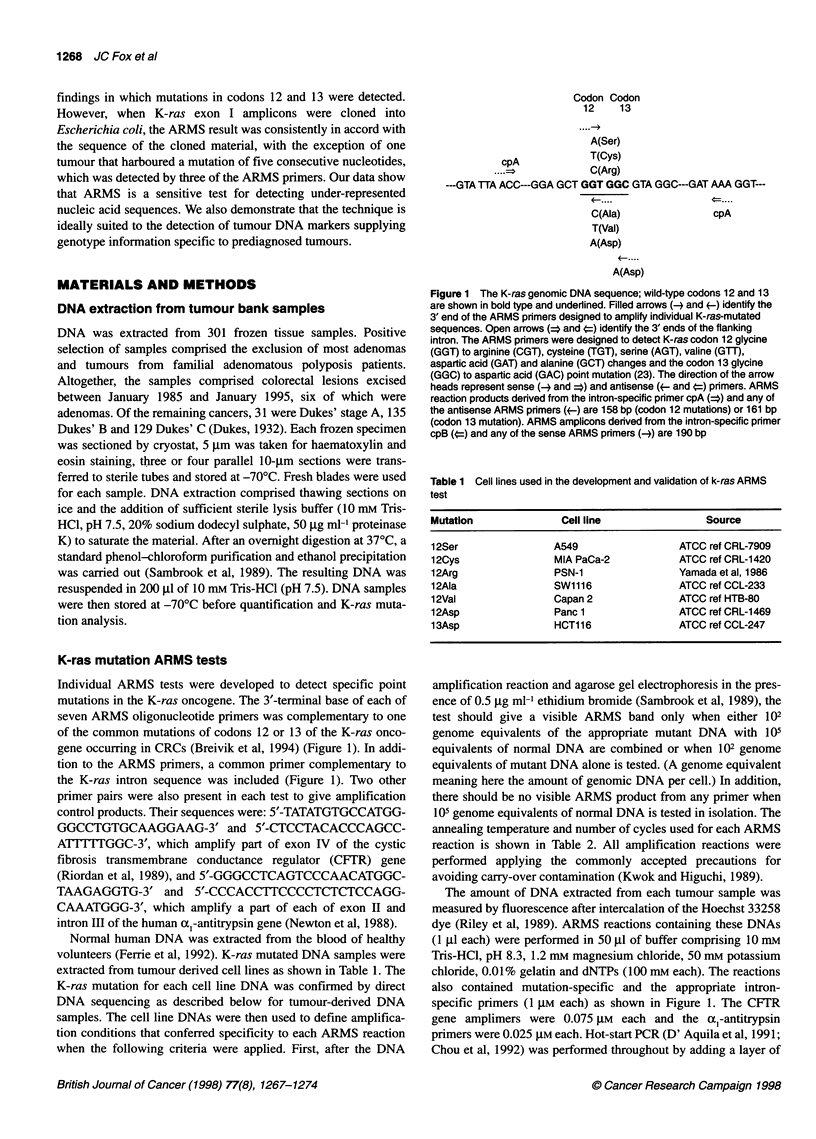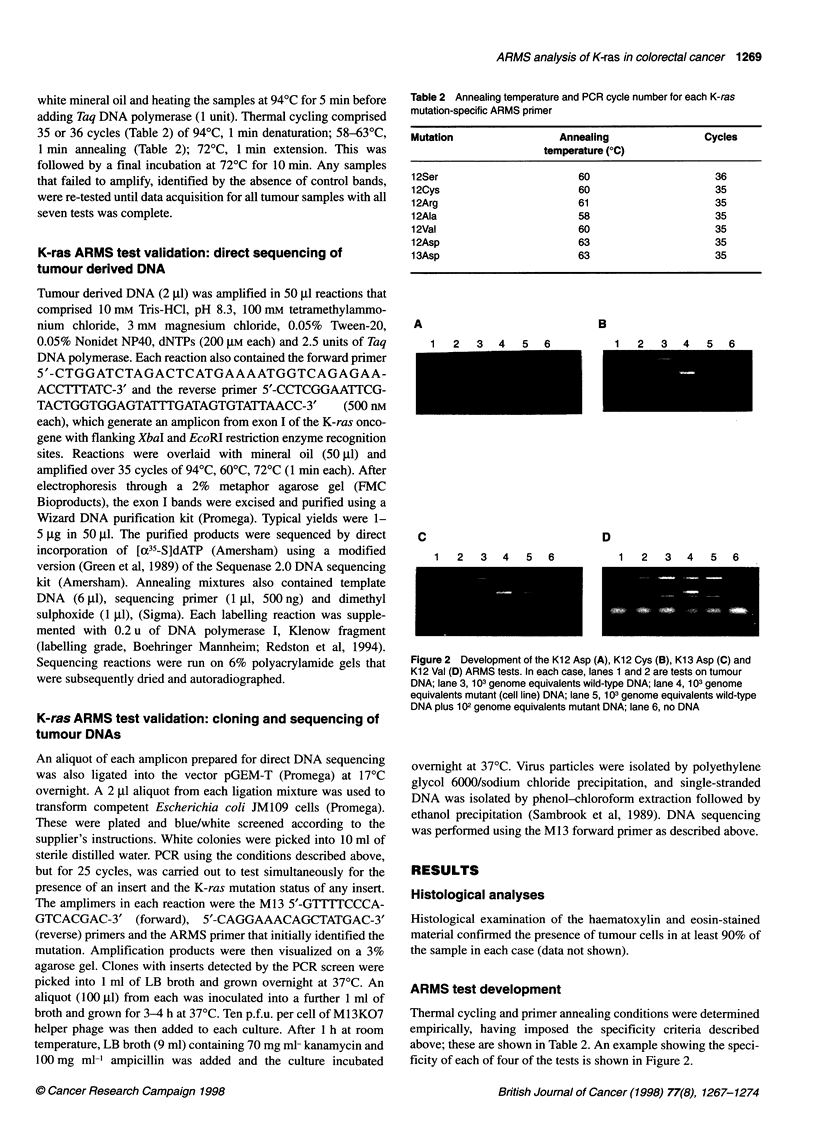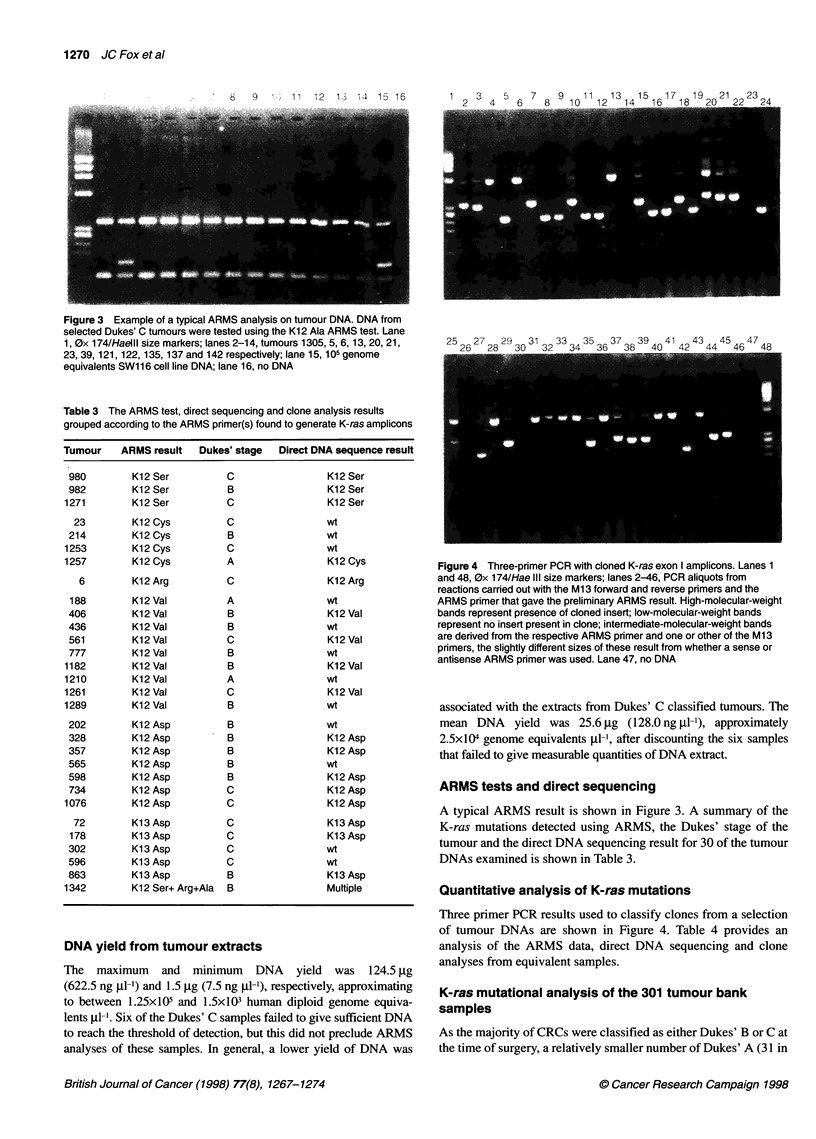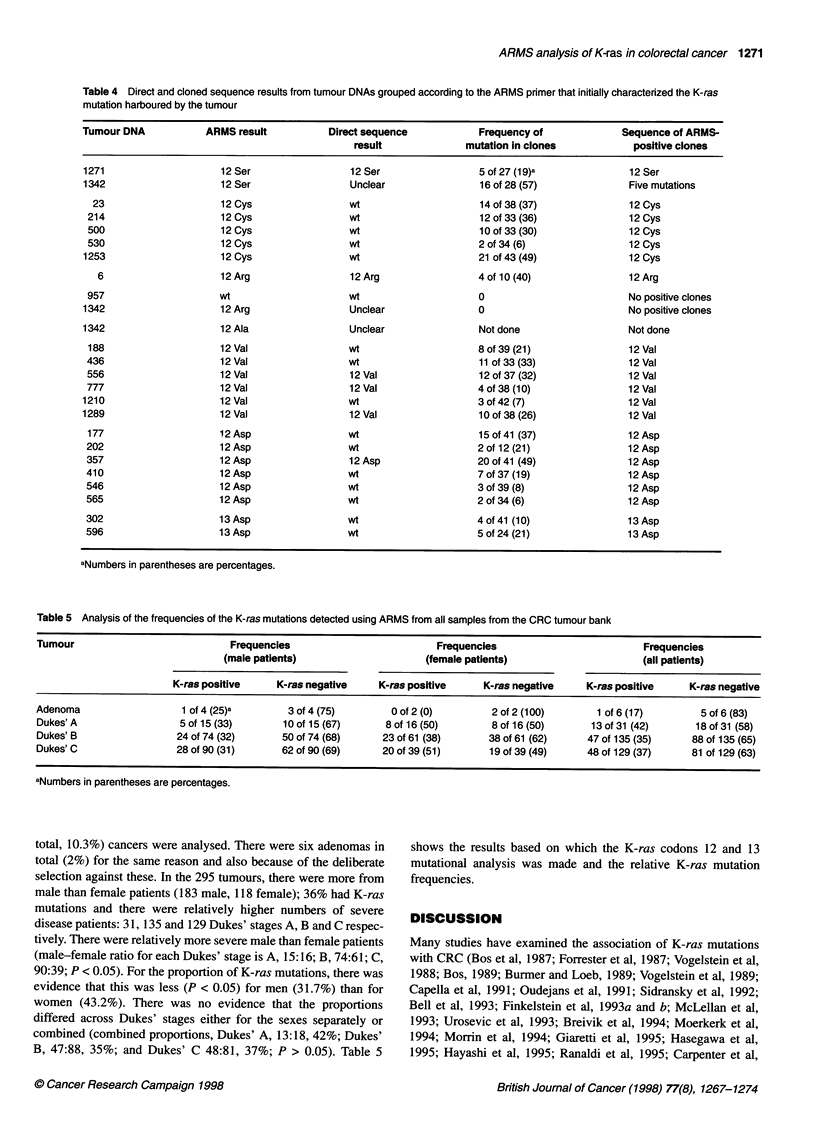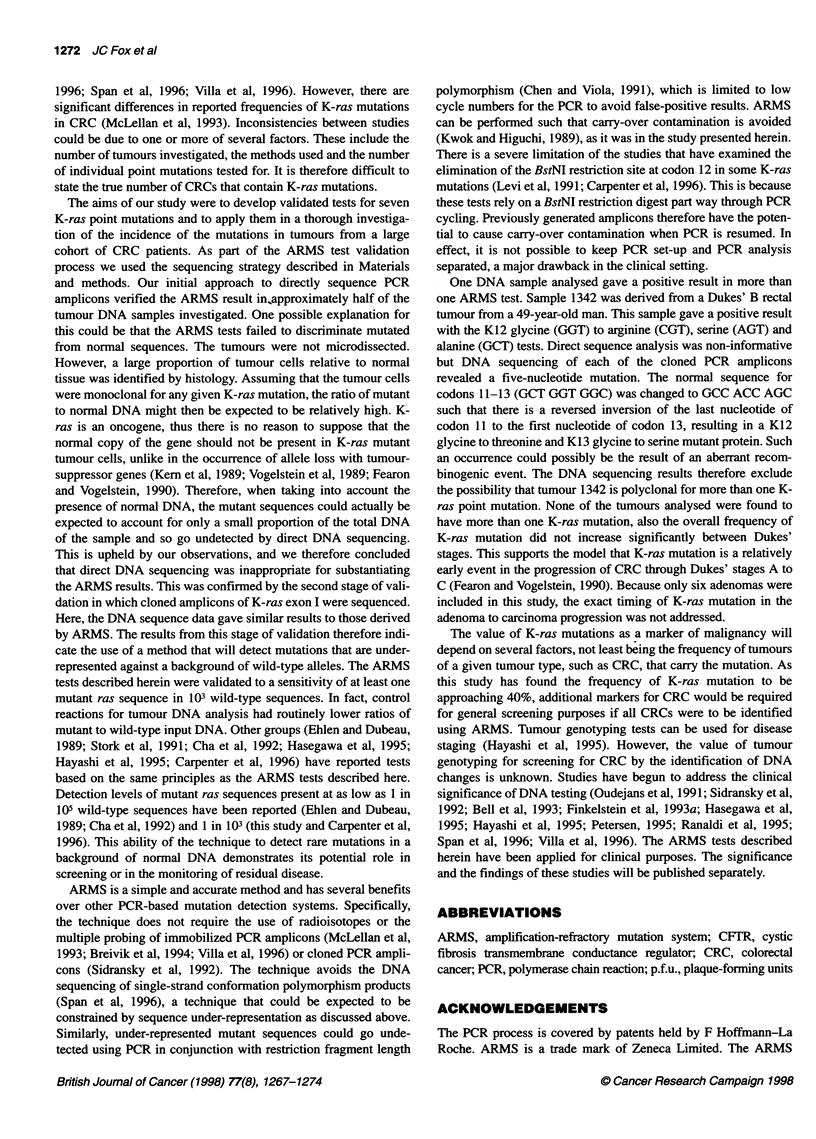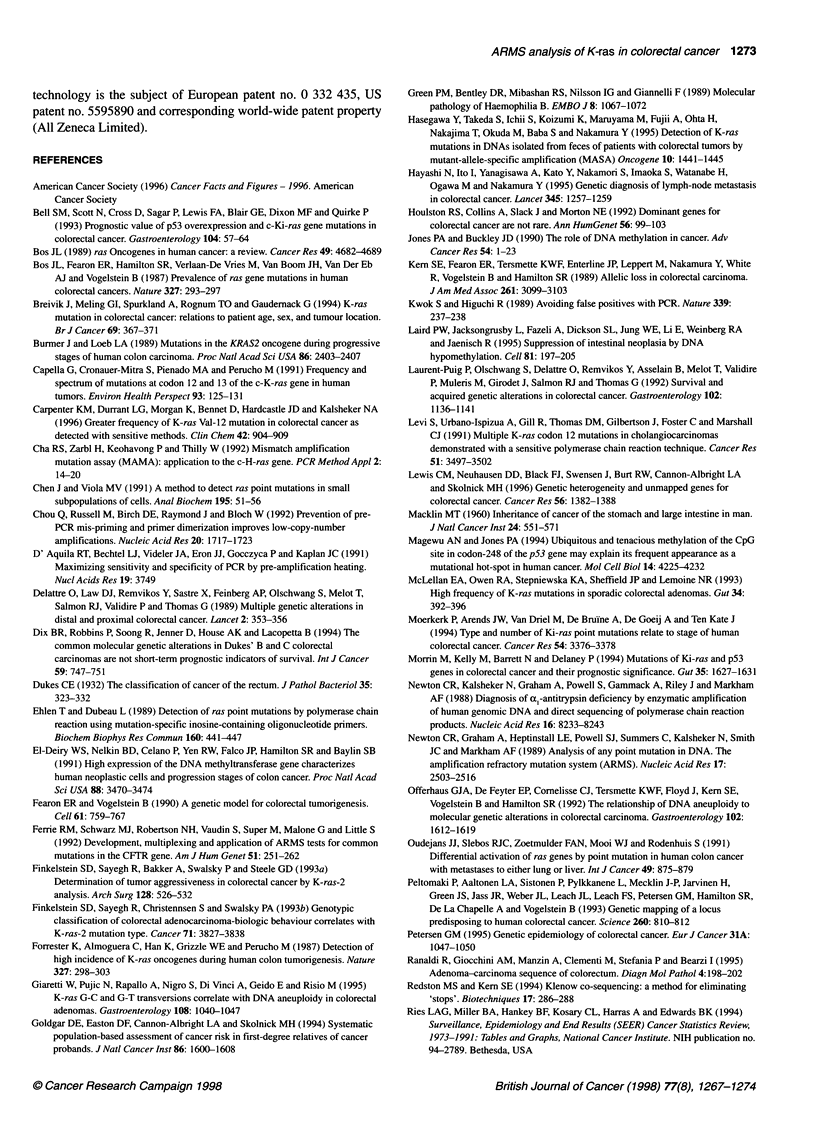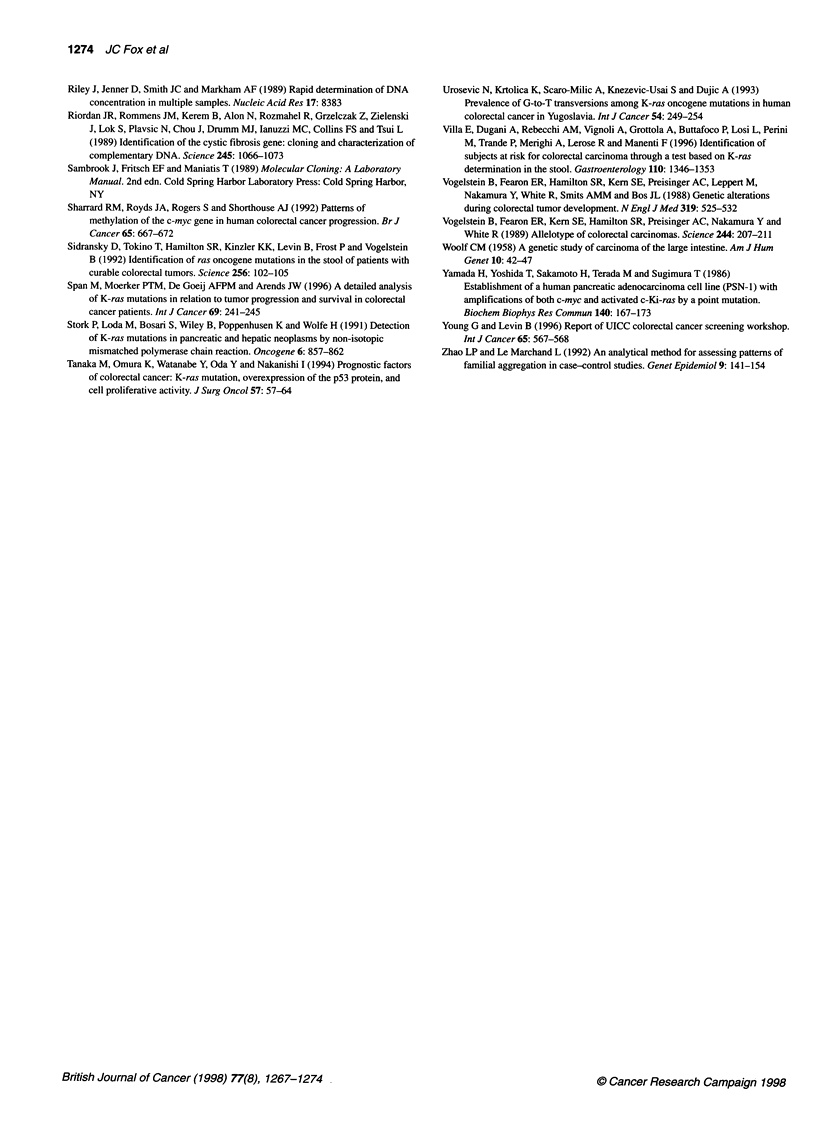# The detection of K-ras mutations in colorectal cancer using the amplification-refractory mutation system.

**DOI:** 10.1038/bjc.1998.212

**Published:** 1998-04

**Authors:** J. C. Fox, J. England, P. White, G. Ellison, K. Callaghan, N. R. Charlesworth, J. Hehir, T. L. McCarthy, J. Smith-Ravin, I. C. Talbot, D. Snary, J. M. Northover, C. R. Newton, S. Little

**Affiliations:** Zeneca Diagnostics, Northwich, Cheshire, UK.

## Abstract

**Images:**


British Journal of Cancer (1998) 77(8), 1267–1274
© 1998 Cancer Research Campaign

### The detection of K-*ras* mutations in colorectal cancer using the amplification-refractory mutation system

**JC Fox[1], J England[2], P White[1], G Ellison[1], K Callaghan[1], NR Charlesworth[1], J Hehir[1], TL McCarthy[1], J Smith-Ravin[2], IC Talbot[2], D Snary[3], JMA Northover[2], CR Newton[1] and S Little[1]**

[1]Zeneca Diagnostics, Gadbrook Park, Northwich, Cheshire CW9 7RA, UK; [2]Imperial Cancer Research Fund, Colorectal Unit, St Mark's Hospital, Northwick Park, Harrow, Middlesex HA1 3UJ, UK; [3]Imperial Cancer Research Fund, Applied Development Laboratory, Dominion House, 59 Bartholomew Close, St Bartholomew's Hospital, London EC1A 7BE, UK

**Summary** A total of 301 colorectal carcinoma (CRC) archival samples were analysed using the amplification-refractory mutation system (ARMS). Each sample was examined to determine the mutation status of codons 12 and 13 of the K-*ras* oncogene. The results from direct DNA sequence analysis carried out on 30 of the samples differed from the ARMS result in almost 50% of the cases as a result of the relative excess of wild-type to mutated DNA sequences. To assess the validity of the ARMS data, the polymerase chain reaction (PCR) was used to generate an amplicon from K-*ras* exon I from 23 of the samples. The PCR amplicons were cloned and sequenced, and the DNA sequence analysis of the cloned material was in agreement with the ARMS results in all but one case. This case represented a tumour that exhibited a five-nucleotide reversed inversion. The cloned sequence data confirm the sensitivity and specificity of the individual ARMS reactions and that it is possible in certain cases to detect additional, more complex, sequence variations.

**Keywords:** amplification-refractory mutation system; diagnosis; mutation; oncogene; screening

Colorectal cancer is the second most common malignancy in the USA (Ries et al, 1994) and is a significant cause of morbidity and mortality world-wide. The American Cancer Society estimated that in 1995 more than 130 000 new cases of CRC would be diagnosed in the USA and that there would be 54 900 deaths from the disease (American Cancer Society, 1995). CRC onset and its progression has been studied extensively at the molecular (Bos et al, 1987; Forrester et al, 1987; Vogelstein et al, 1988; Burmer and Loeb, 1989; Delattre et al, 1989; Kern et al, 1989; Vogelstein et al, 1989; Fearon and Vogelstein, 1990; El-Deiry et al, 1991; Oudejans et al, 1991; Houlston et al, 1992; Laurent-Puig et al, 1992; Offerhaus et al, 1992; Sharrard et al, 1992; Bell et al, 1993; Finkelstein et al, 1993*a* and *b*; McLellan et al, 1993; Peltomaki et al, 1993; Urosevic et al, 1993; Breivik et al, 1994; Dix et al, 1994; Moerkerk et al, 1994; Morrin et al, 1994; Tanaka et al, 1994; Giaretti et al, 1995; Laird et al, 1995; Lewis et al, 1996; Span et al, 1996) and genetic (Woolf et al, 1958; Macklin et al, 1960; Houlston et al, 1992; Zhao and Le Marchand, 1992; Peltomaki et al, 1993; Goldgar et al, 1994; Lewis et al, 1996) levels and there is a commonly accepted model relating tumour grade according to Dukes' stage (Dukes, 1932) to specific DNA changes (Fearon and Vogelstein, 1990).

It has recently been concluded that reliable CRC screening procedures require development and that additional research is needed to identify mutated genes in blood and stool (Young and Levin, 1996). Although there are many examples of nucleic acid changes having potential as tumour markers (Bos et al, 1987; Forrester et al, 1987; Vogelstein et al, 1988; Burmer and Loeb, 1989; Delattre et al, 1989; Kern et al, 1989; Vogelstein et al, 1989; Fearon and Vogelstein, 1990; Jones and Buckley, 1990; Capella et al, 1991; El-Deiry et al, 1991; Oudejans et al, 1991; Stork et al, 1991; Laurent-Puig et al, 1992; Offerhaus et al, 1992; Sharrard et al, 1992; Bell et al, 1993; Finkelstein et al, 1993*a* and *b*; McLellan et al, 1993; Urosevic et al, 1993; Breivik et al, 1994; Dix et al, 1994; Magewu and Jones, 1994; Moerkerk et al, 1994; Morrin et al, 1994; Tanaka et al, 1994; Giaretti et al, 1995; Laird et al, 1995; Span et al, 1996), their value as clinical tools in cancer diagnosis, staging or even screening needs to be demonstrated and two important criteria must be met. First, adequate supplies of nucleic acid must be extracted from the clinical material; second, robust and accurate methods of analysis are required. For reliable tumour genotyping to be useful in disease staging, any test has to be adequately validated and there should be demonstrable benefits over current methods.

A significant proportion of CRCs have mutations in the K-*ras* oncogene (Bos et al, 1987; Forrester et al, 1987; Vogelstein et al, 1988; Burmer and Loeb, 1989; Delattre et al, 1989; Fearon and Vogelstein, 1990; Capella et al, 1991; Oudejans et al, 1991; Offerhaus et al, 1992; Bell et al, 1993; Finkelstein et al, 1993*a* and *b*; McLellan et al, 1993; Peltomaki et al, 1993; Urosevic et al, 1993; Breivik et al, 1994; Moerkerk et al, 1994; Morrin et al, 1994; Tanaka et al, 1994; Giaretti et al, 1995; Span et al, 1996). We report a study using 301 DNA samples extracted from a colorectal tumour bank. In this study, mutations within codons 12 and 13 of the K-*ras* oncogene were investigated using amplification-refractory mutation system (ARMS) (Newton et al, 1989). Direct DNA sequencing (Newton et al, 1988) and sequencing of cloned amplicons were then performed to assess the ARMS test results. In almost half of the cases, the direct sequencing result detected K-*ras* wild-type sequence only, this was in contrast to the ARMS

*Received 18 June 1997*
*Revised 19 August 1997*
*Accepted 4 September 1997*

*Correspondence to:* JC Fox findings in which mutations in codons 12 and 13 were detected. However, when K-*ras* exon I amplicons were cloned into *Escherichia coli*, the ARMS result was consistently in accord with the sequence of the cloned material, with the exception of one tumour that harboured a mutation of five consecutive nucleotides, which was detected by three of the ARMS primers. Our data show that ARMS is a sensitive test for detecting under-represented nucleic acid sequences. We also demonstrate that the technique is ideally suited to the detection of tumour DNA markers supplying genotype information specific to prediagnosed tumours.

### MATERIALS AND METHODS

### DNA extraction from tumour bank samples

DNA was extracted from 301 frozen tissue samples. Positive selection of samples comprised the exclusion of most adenomas and tumours from familial adenomatous polyposis patients. Altogether, the samples comprised colorectal lesions excised between January 1985 and January 1995, six of which were adenomas. Of the remaining cancers, 31 were Dukes' stage A, 135 Dukes' B and 129 Dukes' C (Dukes, 1932). Each frozen specimen was sectioned by cryostat, 5 μm was taken for haematoxylin and eosin staining, three or four parallel 10-μm sections were transferred to sterile tubes and stored at –70°C. Fresh blades were used for each sample. DNA extraction comprised thawing sections on ice and the addition of sufficient sterile lysis buffer (10 mM Tris-HCl, pH 7.5, 20% sodium dodecyl sulphate, 50 μg $ml^{-1}$ proteinase K) to saturate the material. After an overnight digestion at 37°C, a standard phenol–chloroform purification and ethanol precipitation was carried out (Sambrook et al, 1989). The resulting DNA was resuspended in 200 μl of 10 mM Tris-HCl (pH 7.5). DNA samples were then stored at –70°C before quantification and K-*ras* mutation analysis.

**Figure 1** The K-*ras* genomic DNA sequence; wild-type codons 12 and 13 are shown in bold type and underlined. Filled arrows (→ and ←) identify the 3′ end of the ARMS primers designed to amplify individual K-*ras*-mutated sequences. Open arrows (⇒ and ⇐) identify the 3′ ends of the flanking intron. The ARMS primers were designed to detect K-*ras* codon 12 glycine (GGT) to arginine (CGT), cysteine (TGT), serine (AGT), valine (GTT), aspartic acid (GAT) and alanine (GCT) changes and the codon 13 glycine (GGC) to aspartic acid (GAC) point mutation (23). The direction of the arrow heads represent sense (→ and ⇒) and antisense (← and ⇐) primers. ARMS reaction products derived from the intron-specific primer cpA (⇒) and any of the antisense ARMS primers (←) are 158 bp (codon 12 mutations) or 161 bp (codon 13 mutation). ARMS amplicons derived from the intron-specific primer cpB (⇐) and any of the sense ARMS primers (→) are 190 bp

**Table 1** Cell lines used in the development and validation of k-*ras* ARMS test

| Mutation | Cell line | Source |
|---|---|---|
| 12Ser | A549 | ATCC ref CRL-7909 |
| 12Cys | MIA PaCa-2 | ATCC ref CRL-1420 |
| 12Arg | PSN-1 | Yamada et al, 1986 |
| 12Ala | SW1116 | ATCC ref CCL-233 |
| 12Val | Capan 2 | ATCC ref HTB-80 |
| 12Asp | Panc 1 | ATCC ref CRL-1469 |
| 13Asp | HCT116 | ATCC ref CCL-247 |

### K-ras mutation ARMS tests

Individual ARMS tests were developed to detect specific point mutations in the K-*ras* oncogene. The 3′-terminal base of each of seven ARMS oligonucleotide primers was complementary to one of the common mutations of codons 12 or 13 of the K-*ras* oncogene occurring in CRCs (Breivik et al, 1994) (Figure 1). In addition to the ARMS primers, a common primer complementary to the K-*ras* intron sequence was included (Figure 1). Two other primer pairs were also present in each test to give amplification control products. Their sequences were: 5′-TATATGTGCCATGG-GGCCTGTGCAAGGAAG-3′ and 5′-CTCCTACACCCAGCC-ATTTTTGGC-3′, which amplify part of exon IV of the cystic fibrosis transmembrane conductance regulator (CFTR) gene (Riordan et al, 1989), and 5′-GGGCCTCAGTCCCAACATGGC-TAAGAGGTG-3′ and 5′-CCCACCTTCCCCTCTCTCCAGG-CAAATGGG-3′, which amplify a part of each of exon II and intron III of the human $\alpha_1$-antitrypsin gene (Newton et al, 1988).

Normal human DNA was extracted from the blood of healthy volunteers (Ferrie et al, 1992). K-*ras* mutated DNA samples were extracted from tumour derived cell lines as shown in Table 1. The K-*ras* mutation for each cell line DNA was confirmed by direct DNA sequencing as described below for tumour-derived DNA samples. The cell line DNAs were then used to define amplification conditions that conferred specificity to each ARMS reaction when the following criteria were applied. First, after the DNA amplification reaction and agarose gel electrophoresis in the presence of 0.5 μg $ml^{-1}$ ethidium bromide (Sambrook et al, 1989), the test should give a visible ARMS band only when either $10^2$ genome equivalents of the appropriate mutant DNA with $10^5$ equivalents of normal DNA are combined or when $10^2$ genome equivalents of mutant DNA alone is tested. (A genome equivalent meaning here the amount of genomic DNA per cell.) In addition, there should be no visible ARMS product from any primer when $10^5$ genome equivalents of normal DNA is tested in isolation. The annealing temperature and number of cycles used for each ARMS reaction is shown in Table 2. All amplification reactions were performed applying the commonly accepted precautions for avoiding carry-over contamination (Kwok and Higuchi, 1989).

The amount of DNA extracted from each tumour sample was measured by fluorescence after intercalation of the Hoechst 33258 dye (Riley et al, 1989). ARMS reactions containing these DNAs (1 μl each) were performed in 50 μl of buffer comprising 10 mM Tris-HCl, pH 8.3, 1.2 mM magnesium chloride, 50 mM potassium chloride, 0.01% gelatin and dNTPs (100 mM each). The reactions also contained mutation-specific and the appropriate intron-specific primers (1 μM each) as shown in Figure 1. The CFTR gene amplimers were 0.075 μM each and the $\alpha_1$-antitrypsin primers were 0.025 μM each. Hot-start PCR (D' Aquila et al, 1991; Chou et al, 1992) was performed throughout by adding a layer of

© Cancer Research Campaign 1998

white mineral oil and heating the samples at 94°C for 5 min before adding *Taq* DNA polymerase (1 unit). Thermal cycling comprised 35 or 36 cycles (Table 2) of 94°C, 1 min denaturation; 58–63°C, 1 min annealing (Table 2); 72°C, 1 min extension. This was followed by a final incubation at 72°C for 10 min. Any samples that failed to amplify, identified by the absence of control bands, were re-tested until data acquisition for all tumour samples with all seven tests was complete.

### K-ras ARMS test validation: direct sequencing of tumour derived DNA

Tumour derived DNA (2 µl) was amplified in 50 µl reactions that comprised 10 mM Tris-HCl, pH 8.3, 100 mM tetramethylammonium chloride, 3 mM magnesium chloride, 0.05% Tween-20, 0.05% Nonidet NP40, dNTPs (200 µM each) and 2.5 units of *Taq* DNA polymerase. Each reaction also contained the forward primer 5′-CTGGATCTAGACTCATGAAAATGGTCAGAGAA-ACCTTTATC-3′ and the reverse primer 5′-CCTCGGAATTCG-TACTGGTGGAGTATTTGATAGTGTATTAACC-3′ (500 nM each), which generate an amplicon from exon I of the K-*ras* oncogene with flanking *Xba*I and *Eco*RI restriction enzyme recognition sites. Reactions were overlaid with mineral oil (50 µl) and amplified over 35 cycles of 94°C, 60°C, 72°C (1 min each). After electrophoresis through a 2% metaphor agarose gel (FMC Bioproducts), the exon I bands were excised and purified using a Wizard DNA purification kit (Promega). Typical yields were 1–5 µg in 50 µl. The purified products were sequenced by direct incorporation of [$\alpha^{35}$-S]dATP (Amersham) using a modified version (Green et al, 1989) of the Sequenase 2.0 DNA sequencing kit (Amersham). Annealing mixtures also contained template DNA (6 µl), sequencing primer (1 µl, 500 ng) and dimethyl sulphoxide (1 µl), (Sigma). Each labelling reaction was supplemented with 0.2 u of DNA polymerase I, Klenow fragment (labelling grade, Boehringer Mannheim; Redston et al, 1994). Sequencing reactions were run on 6% polyacrylamide gels that were subsequently dried and autoradiographed.

### K-*ras* ARMS test validation: cloning and sequencing of tumour DNAs

An aliquot of each amplicon prepared for direct DNA sequencing was also ligated into the vector pGEM-T (Promega) at 17°C overnight. A 2 µl aliquot from each ligation mixture was used to transform competent *Escherichia coli* JM109 cells (Promega). These were plated and blue/white screened according to the supplier's instructions. White colonies were picked into 10 ml of sterile distilled water. PCR using the conditions described above, but for 25 cycles, was carried out to test simultaneously for the presence of an insert and the K-*ras* mutation status of any insert. The amplimers in each reaction were the M13 5′-GTTTTCCCA-GTCACGAC-3′ (forward), 5′-CAGGAAACAGCTATGAC-3′ (reverse) primers and the ARMS primer that initially identified the mutation. Amplification products were then visualized on a 3% agarose gel. Clones with inserts detected by the PCR screen were picked into 1 ml of LB broth and grown overnight at 37°C. An aliquot (100 µl) from each was inoculated into a further 1 ml of broth and grown for 3–4 h at 37°C. Ten p.f.u. per cell of M13KO7 helper phage was then added to each culture. After 1 h at room temperature, LB broth (9 ml) containing 70 mg ml$^{-}$ kanamycin and 100 mg ml$^{-1}$ ampicillin was added and the culture incubated overnight at 37°C. Virus particles were isolated by polyethylene glycol 6000/sodium chloride precipitation, and single-stranded DNA was isolated by phenol–chloroform extraction followed by ethanol precipitation (Sambrook et al, 1989). DNA sequencing was performed using the M13 forward primer as described above.

**Table 2** Annealing temperature and PCR cycle number for each K-*ras* mutation-specific ARMS primer

| Mutation | Annealing temperature (°C) | Cycles |
|---|---|---|
| 12Ser | 60 | 36 |
| 12Cys | 60 | 35 |
| 12Arg | 61 | 35 |
| 12Ala | 58 | 35 |
| 12Val | 60 | 35 |
| 12Asp | 63 | 35 |
| 13Asp | 63 | 35 |

**Figure 2** Development of the K12 Asp (**A**), K12 Cys (**B**), K13 Asp (**C**) and K12 Val (**D**) ARMS tests. In each case, lanes 1 and 2 are tests on tumour DNA; lane 3, $10^3$ genome equivalents wild-type DNA; lane 4, $10^3$ genome equivalents mutant (cell line) DNA; lane 5, $10^3$ genome equivalents wild-type DNA plus $10^2$ genome equivalents mutant DNA; lane 6, no DNA

### RESULTS

### Histological analyses

Histological examination of the haematoxylin and eosin-stained material confirmed the presence of tumour cells in at least 90% of the sample in each case (data not shown).

### ARMS test development

Thermal cycling and primer annealing conditions were determined empirically, having imposed the specificity criteria described above; these are shown in Table 2. An example showing the specificity of each of four of the tests is shown in Figure 2.

© Cancer Research Campaign 1998

**Figure 3** Example of a typical ARMS analysis on tumour DNA. DNA from selected Dukes' C tumours were tested using the K12 Ala ARMS test. Lane 1, Øx 174/*Hae*III size markers; lanes 2–14, tumours 1305, 5, 6, 13, 20, 21, 23, 39, 121, 122, 135, 137 and 142 respectively; lane 15, $10^5$ genome equivalents SW116 cell line DNA; lane 16, no DNA

**Table 3** The ARMS test, direct sequencing and clone analysis results grouped according to the ARMS primer(s) found to generate K-*ras* amplicons

| Tumour | ARMS result | Dukes' stage | Direct DNA sequence result |
|---|---|---|---|
| 980 | K12 Ser | C | K12 Ser |
| 982 | K12 Ser | B | K12 Ser |
| 1271 | K12 Ser | C | K12 Ser |
| 23 | K12 Cys | C | wt |
| 214 | K12 Cys | B | wt |
| 1253 | K12 Cys | C | wt |
| 1257 | K12 Cys | A | K12 Cys |
| 6 | K12 Arg | C | K12 Arg |
| 188 | K12 Val | A | wt |
| 406 | K12 Val | B | K12 Val |
| 436 | K12 Val | B | wt |
| 561 | K12 Val | C | K12 Val |
| 777 | K12 Val | B | wt |
| 1182 | K12 Val | B | K12 Val |
| 1210 | K12 Val | A | wt |
| 1261 | K12 Val | C | K12 Val |
| 1289 | K12 Val | B | wt |
| 202 | K12 Asp | B | wt |
| 328 | K12 Asp | B | K12 Asp |
| 357 | K12 Asp | B | K12 Asp |
| 565 | K12 Asp | B | wt |
| 598 | K12 Asp | B | K12 Asp |
| 734 | K12 Asp | C | K12 Asp |
| 1076 | K12 Asp | C | K12 Asp |
| 72 | K13 Asp | C | K13 Asp |
| 178 | K13 Asp | C | K13 Asp |
| 302 | K13 Asp | C | wt |
| 596 | K13 Asp | C | wt |
| 863 | K13 Asp | B | K13 Asp |
| 1342 | K12 Ser+ Arg+Ala | B | Multiple |

### DNA yield from tumour extracts

The maximum and minimum DNA yield was 124.5 μg (622.5 ng $\mu l^{-1}$) and 1.5 μg (7.5 ng $\mu l^{-1}$), respectively, approximating to between $1.25 \times 10^5$ and $1.5 \times 10^3$ human diploid genome equivalents $\mu l^{-1}$. Six of the Dukes' C samples failed to give sufficient DNA to reach the threshold of detection, but this did not preclude ARMS analyses of these samples. In general, a lower yield of DNA was associated with the extracts from Dukes' C classified tumours. The mean DNA yield was 25.6 μg (128.0 ng $\mu l^{-1}$), approximately $2.5 \times 10^4$ genome equivalents $\mu l^{-1}$, after discounting the six samples that failed to give measurable quantities of DNA extract.

**Figure 4** Three-primer PCR with cloned K-*ras* exon I amplicons. Lanes 1 and 48, Øx 174/*Hae* III size markers; lanes 2–46, PCR aliquots from reactions carried out with the M13 forward and reverse primers and the ARMS primer that gave the preliminary ARMS result. High-molecular-weight bands represent presence of cloned insert; low-molecular-weight bands represent no insert present in clone; intermediate-molecular-weight bands are derived from the respective ARMS primer and one or other of the M13 primers, the slightly different sizes of these result from whether a sense or antisense ARMS primer was used. Lane 47, no DNA

### ARMS tests and direct sequencing

A typical ARMS result is shown in Figure 3. A summary of the K-*ras* mutations detected using ARMS, the Dukes' stage of the tumour and the direct DNA sequencing result for 30 of the tumour DNAs examined is shown in Table 3.

### Quantitative analysis of K-*ras* mutations

Three primer PCR results used to classify clones from a selection of tumour DNAs are shown in Figure 4. Table 4 provides an analysis of the ARMS data, direct DNA sequencing and clone analyses from equivalent samples.

### K-*ras* mutational analysis of the 301 tumour bank samples

As the majority of CRCs were classified as either Dukes' B or C at the time of surgery, a relatively smaller number of Dukes' A (31 in

© Cancer Research Campaign 1998

**Table 4** Direct and cloned sequence results from tumour DNAs grouped according to the ARMS primer that initially characterized the K-*ras* mutation harboured by the tumour

| Tumour DNA | ARMS result | Direct sequence result | Frequency of mutation in clones | Sequence of ARMS-positive clones |
|---|---|---|---|---|
| 1271 | 12 Ser | 12 Ser | 5 of 27 (19)[a] | 12 Ser |
| 1342 | 12 Ser | Unclear | 16 of 28 (57) | Five mutations |
| 23 | 12 Cys | wt | 14 of 38 (37) | 12 Cys |
| 214 | 12 Cys | wt | 12 of 33 (36) | 12 Cys |
| 500 | 12 Cys | wt | 10 of 33 (30) | 12 Cys |
| 530 | 12 Cys | wt | 2 of 34 (6) | 12 Cys |
| 1253 | 12 Cys | wt | 21 of 43 (49) | 12 Cys |
| 6 | 12 Arg | 12 Arg | 4 of 10 (40) | 12 Arg |
| 957 | wt | wt | 0 | No positive clones |
| 1342 | 12 Arg | Unclear | 0 | No positive clones |
| 1342 | 12 Ala | Unclear | Not done | Not done |
| 188 | 12 Val | wt | 8 of 39 (21) | 12 Val |
| 436 | 12 Val | wt | 11 of 33 (33) | 12 Val |
| 556 | 12 Val | 12 Val | 12 of 37 (32) | 12 Val |
| 777 | 12 Val | 12 Val | 4 of 38 (10) | 12 Val |
| 1210 | 12 Val | wt | 3 of 42 (7) | 12 Val |
| 1289 | 12 Val | 12 Val | 10 of 38 (26) | 12 Val |
| 177 | 12 Asp | wt | 15 of 41 (37) | 12 Asp |
| 202 | 12 Asp | wt | 2 of 12 (21) | 12 Asp |
| 357 | 12 Asp | 12 Asp | 20 of 41 (49) | 12 Asp |
| 410 | 12 Asp | wt | 7 of 37 (19) | 12 Asp |
| 546 | 12 Asp | wt | 3 of 39 (8) | 12 Asp |
| 565 | 12 Asp | wt | 2 of 34 (6) | 12 Asp |
| 302 | 13 Asp | wt | 4 of 41 (10) | 13 Asp |
| 596 | 13 Asp | wt | 5 of 24 (21) | 13 Asp |

[a]Numbers in parentheses are percentages.

**Table 5** Analysis of the frequencies of the K-*ras* mutations detected using ARMS from all samples from the CRC tumour bank

| Tumour | Frequencies (male patients) | | Frequencies (female patients) | | Frequencies (all patients) | |
|---|---|---|---|---|---|---|
| | K-*ras* positive | K-*ras* negative | K-*ras* positive | K-*ras* negative | K-*ras* positive | K-*ras* negative |
| Adenoma | 1 of 4 (25)[a] | 3 of 4 (75) | 0 of 2 (0) | 2 of 2 (100) | 1 of 6 (17) | 5 of 6 (83) |
| Dukes' A | 5 of 15 (33) | 10 of 15 (67) | 8 of 16 (50) | 8 of 16 (50) | 13 of 31 (42) | 18 of 31 (58) |
| Dukes' B | 24 of 74 (32) | 50 of 74 (68) | 23 of 61 (38) | 38 of 61 (62) | 47 of 135 (35) | 88 of 135 (65) |
| Dukes' C | 28 of 90 (31) | 62 of 90 (69) | 20 of 39 (51) | 19 of 39 (49) | 48 of 129 (37) | 81 of 129 (63) |

[a]Numbers in parentheses are percentages.

total, 10.3%) cancers were analysed. There were six adenomas in total (2%) for the same reason and also because of the deliberate selection against these. In the 295 tumours, there were more from male than female patients (183 male, 118 female); 36% had K-*ras* mutations and there were relatively higher numbers of severe disease patients: 31, 135 and 129 Dukes' stages A, B and C respectively. There were relatively more severe male than female patients (male–female ratio for each Dukes' stage is A, 15:16; B, 74:61; C, 90:39; $P < 0.05$). For the proportion of K-*ras* mutations, there was evidence that this was less ($P < 0.05$) for men (31.7%) than for women (43.2%). There was no evidence that the proportions differed across Dukes' stages either for the sexes separately or combined (combined proportions, Dukes' A, 13:18, 42%; Dukes' B, 47:88, 35%; and Dukes' C 48:81, 37%; $P > 0.05$). Table 5 shows the results based on which the K-*ras* codons 12 and 13 mutational analysis was made and the relative K-*ras* mutation frequencies.

### DISCUSSION

Many studies have examined the association of K-*ras* mutations with CRC (Bos et al, 1987; Forrester et al, 1987; Vogelstein et al, 1988; Bos, 1989; Burmer and Loeb, 1989; Vogelstein et al, 1989; Capella et al, 1991; Oudejans et al, 1991; Sidransky et al, 1992; Bell et al, 1993; Finkelstein et al, 1993*a* and *b*; McLellan et al, 1993; Urosevic et al, 1993; Breivik et al, 1994; Moerkerk et al, 1994; Morrin et al, 1994; Giaretti et al, 1995; Hasegawa et al, 1995; Hayashi et al, 1995; Ranaldi et al, 1995; Carpenter et al,

© Cancer Research Campaign 1998

1996; Span et al, 1996; Villa et al, 1996). However, there are significant differences in reported frequencies of K-*ras* mutations in CRC (McLellan et al, 1993). Inconsistencies between studies could be due to one or more of several factors. These include the number of tumours investigated, the methods used and the number of individual point mutations tested for. It is therefore difficult to state the true number of CRCs that contain K-*ras* mutations.

The aims of our study were to develop validated tests for seven K-*ras* point mutations and to apply them in a thorough investigation of the incidence of the mutations in tumours from a large cohort of CRC patients. As part of the ARMS test validation process we used the sequencing strategy described in Materials and methods. Our initial approach to directly sequence PCR amplicons verified the ARMS result in approximately half of the tumour DNA samples investigated. One possible explanation for this could be that the ARMS tests failed to discriminate mutated from normal sequences. The tumours were not microdissected. However, a large proportion of tumour cells relative to normal tissue was identified by histology. Assuming that the tumour cells were monoclonal for any given K-*ras* mutation, the ratio of mutant to normal DNA might then be expected to be relatively high. K-*ras* is an oncogene, thus there is no reason to suppose that the normal copy of the gene should not be present in K-*ras* mutant tumour cells, unlike in the occurrence of allele loss with tumour-suppressor genes (Kern et al, 1989; Vogelstein et al, 1989; Fearon and Vogelstein, 1990). Therefore, when taking into account the presence of normal DNA, the mutant sequences could actually be expected to account for only a small proportion of the total DNA of the sample and so go undetected by direct DNA sequencing. This is upheld by our observations, and we therefore concluded that direct DNA sequencing was inappropriate for substantiating the ARMS results. This was confirmed by the second stage of validation in which cloned amplicons of K-*ras* exon I were sequenced. Here, the DNA sequence data gave similar results to those derived by ARMS. The results from this stage of validation therefore indicate the use of a method that will detect mutations that are under-represented against a background of wild-type alleles. The ARMS tests described herein were validated to a sensitivity of at least one mutant *ras* sequence in $10^3$ wild-type sequences. In fact, control reactions for tumour DNA analysis had routinely lower ratios of mutant to wild-type input DNA. Other groups (Ehlen and Dubeau, 1989; Stork et al, 1991; Cha et al, 1992; Hasegawa et al, 1995; Hayashi et al, 1995; Carpenter et al, 1996) have reported tests based on the same principles as the ARMS tests described here. Detection levels of mutant *ras* sequences present at as low as 1 in $10^5$ wild-type sequences have been reported (Ehlen and Dubeau, 1989; Cha et al, 1992) and 1 in $10^3$ (this study and Carpenter et al, 1996). This ability of the technique to detect rare mutations in a background of normal DNA demonstrates its potential role in screening or in the monitoring of residual disease.

ARMS is a simple and accurate method and has several benefits over other PCR-based mutation detection systems. Specifically, the technique does not require the use of radioisotopes or the multiple probing of immobilized PCR amplicons (McLellan et al, 1993; Breivik et al, 1994; Villa et al, 1996) or cloned PCR amplicons (Sidransky et al, 1992). The technique avoids the DNA sequencing of single-strand conformation polymorphism products (Span et al, 1996), a technique that could be expected to be constrained by sequence under-representation as discussed above. Similarly, under-represented mutant sequences could go undetected using PCR in conjunction with restriction fragment length polymorphism (Chen and Viola, 1991), which is limited to low cycle numbers for the PCR to avoid false-positive results. ARMS can be performed such that carry-over contamination is avoided (Kwok and Higuchi, 1989), as it was in the study presented herein. There is a severe limitation of the studies that have examined the elimination of the *Bst*NI restriction site at codon 12 in some K-*ras* mutations (Levi et al, 1991; Carpenter et al, 1996). This is because these tests rely on a *Bst*NI restriction digest part way through PCR cycling. Previously generated amplicons therefore have the potential to cause carry-over contamination when PCR is resumed. In effect, it is not possible to keep PCR set-up and PCR analysis separated, a major drawback in the clinical setting.

One DNA sample analysed gave a positive result in more than one ARMS test. Sample 1342 was derived from a Dukes' B rectal tumour from a 49-year-old man. This sample gave a positive result with the K12 glycine (GGT) to arginine (CGT), serine (AGT) and alanine (GCT) tests. Direct sequence analysis was non-informative but DNA sequencing of each of the cloned PCR amplicons revealed a five-nucleotide mutation. The normal sequence for codons 11–13 (GCT GGT GGC) was changed to GCC ACC AGC such that there is a reversed inversion of the last nucleotide of codon 11 to the first nucleotide of codon 13, resulting in a K12 glycine to threonine and K13 glycine to serine mutant protein. Such an occurrence could possibly be the result of an aberrant recombinogenic event. The DNA sequencing results therefore exclude the possibility that tumour 1342 is polyclonal for more than one K-*ras* point mutation. None of the tumours analysed were found to have more than one K-*ras* mutation, also the overall frequency of K-*ras* mutation did not increase significantly between Dukes' stages. This supports the model that K-*ras* mutation is a relatively early event in the progression of CRC through Dukes' stages A to C (Fearon and Vogelstein, 1990). Because only six adenomas were included in this study, the exact timing of K-*ras* mutation in the adenoma to carcinoma progression was not addressed.

The value of K-*ras* mutations as a marker of malignancy will depend on several factors, not least being the frequency of tumours of a given tumour type, such as CRC, that carry the mutation. As this study has found the frequency of K-*ras* mutation to be approaching 40%, additional markers for CRC would be required for general screening purposes if all CRCs were to be identified using ARMS. Tumour genotyping tests can be used for disease staging (Hayashi et al, 1995). However, the value of tumour genotyping for screening for CRC by the identification of DNA changes is unknown. Studies have begun to address the clinical significance of DNA testing (Oudejans et al, 1991; Sidransky et al, 1992; Bell et al, 1993; Finkelstein et al, 1993*a*; Hasegawa et al, 1995; Hayashi et al, 1995; Petersen, 1995; Ranaldi et al, 1995; Span et al, 1996; Villa et al, 1996). The ARMS tests described herein have been applied for clinical purposes. The significance and the findings of these studies will be published separately.

### ABBREVIATIONS

ARMS, amplification-refractory mutation system; CFTR, cystic fibrosis transmembrane conductance regulator; CRC, colorectal cancer; PCR, polymerase chain reaction; p.f.u., plaque-forming units

### ACKNOWLEDGEMENTS

The PCR process is covered by patents held by F Hoffmann–La Roche. ARMS is a trade mark of Zeneca Limited. The ARMS

© Cancer Research Campaign 1998

technology is the subject of European patent no. 0 332 435, US patent no. 5595890 and corresponding world-wide patent property (All Zeneca Limited).

### REFERENCES

American Cancer Society (1996) *Cancer Facts and Figures – 1996*. American Cancer Society

Bell SM, Scott N, Cross D, Sagar P, Lewis FA, Blair GE, Dixon MF and Quirke P (1993) Prognostic value of p53 overexpression and c-Ki-*ras* gene mutations in colorectal cancer. *Gastroenterology* **104**: 57–64

Bos JL (1989) *ras* Oncogenes in human cancer: a review. *Cancer Res* **49**: 4682–4689

Bos JL, Fearon ER, Hamilton SR, Verlaan-De Vries M, Van Boom JH, Van Der Eb AJ and Vogelstein B (1987) Prevalence of *ras* gene mutations in human colorectal cancers. *Nature* **327**: 293–297

Breivik J, Meling GI, Spurkland A, Rognum TO and Gaudernack G (1994) K-*ras* mutation in colorectal cancer: relations to patient age, sex, and tumour location. *Br J Cancer* **69**: 367–371

Burmer J and Loeb LA (1989) Mutations in the *KRAS2* oncogene during progressive stages of human colon carcinoma. *Proc Natl Acad Sci USA* **86**: 2403–2407

Capella G, Cronauer-Mitra S, Pienado MA and Perucho M (1991) Frequency and spectrum of mutations at codon 12 and 13 of the c-K-*ras* gene in human tumors. *Environ Health Perspect* **93**: 125–131

Carpenter KM, Durrant LG, Morgan K, Bennet D, Hardcastle JD and Kalsheker NA (1996) Greater frequency of K-*ras* Val-12 mutation in colorectal cancer as detected with sensitive methods. *Clin Chem* **42**: 904–909

Cha RS, Zarbl H, Keohavong P and Thilly W (1992) Mismatch amplification mutation assay (MAMA): application to the c-H-*ras* gene. *PCR Method Appl* **2**: 14–20

Chen J and Viola MV (1991) A method to detect *ras* point mutations in small subpopulations of cells. *Anal Biochem* **195**: 51–56

Chou Q, Russell M, Birch DE, Raymond J and Bloch W (1992) Prevention of pre-PCR mis-priming and primer dimerization improves low-copy-number amplifications. *Nucleic Acid Res* **20**: 1717–1723

D' Aquila RT, Bechtel LJ, Videler JA, Eron JJ, Gocczyca P and Kaplan JC (1991) Maximizing sensitivity and specificity of PCR by pre-amplification heating. *Nucl Acids Res* **19**: 3749

Delattre O, Law DJ, Remvikos Y, Sastre X, Feinberg AP, Olschwang S, Melot T, Salmon RJ, Validire P and Thomas G (1989) Multiple genetic alterations in distal and proximal colorectal cancer. *Lancet* **2**: 353–356

Dix BR, Robbins P, Soong R, Jenner D, House AK and Lacopetta B (1994) The common molecular genetic alterations in Dukes' B and C colorectal carcinomas are not short-term prognostic indicators of survival. *Int J Cancer* **59**: 747–751

Dukes CE (1932) The classification of cancer of the rectum. *J Pathol Bacteriol* **35**: 323–332

Ehlen T and Dubeau L (1989) Detection of *ras* point mutations by polymerase chain reaction using mutation-specific inosine-containing oligonucleotide primers. *Biochem Biophys Res Commun* **160**: 441–447

El-Deiry WS, Nelkin BD, Celano P, Yen RW, Falco JP, Hamilton SR and Baylin SB (1991) High expression of the DNA methyltransferase gene characterizes human neoplastic cells and progression stages of colon cancer. *Proc Natl Acad Sci USA* **88**: 3470–3474

Fearon ER and Vogelstein B (1990) A genetic model for colorectal tumorigenesis. *Cell* **61**: 759–767

Ferrie RM, Schwarz MJ, Robertson NH, Vaudin S, Super M, Malone G and Little S (1992) Development, multiplexing and application of ARMS tests for common mutations in the CFTR gene. *Am J Hum Genet* **51**: 251–262

Finkelstein SD, Sayegh R, Bakker A, Swalsky P and Steele GD (1993*a*) Determination of tumor aggressiveness in colorectal cancer by K-*ras*-2 analysis. *Arch Surg* **128**: 526–532

Finkelstein SD, Sayegh R, Christennsen S and Swalsky PA (1993*b*) Genotypic classification of colorectal adenocarcinoma-biologic behaviour correlates with K-*ras*-2 mutation type. *Cancer* **71**: 3827–3838

Forrester K, Almoguera C, Han K, Grizzle WE and Perucho M (1987) Detection of high incidence of K-*ras* oncogenes during human colon tumorigenesis. *Nature* **327**: 298–303

Giaretti W, Pujic N, Rapallo A, Nigro S, Di Vinci A, Geido E and Risio M (1995) K-*ras* G-C and G-T transversions correlate with DNA aneuploidy in colorectal adenomas. *Gastroenterology* **108**: 1040–1047

Goldgar DE, Easton DF, Cannon-Albright LA and Skolnick MH (1994) Systematic population-based assessment of cancer risk in first-degree relatives of cancer probands. *J Natl Cancer Inst* **86**: 1600–1608

Green PM, Bentley DR, Mibashan RS, Nilsson IG and Giannelli F (1989) Molecular pathology of Haemophilia B. *EMBO J* **8**: 1067–1072

Hasegawa Y, Takeda S, Ichii S, Koizumi K, Maruyama M, Fujii A, Ohta H, Nakajima T, Okuda M, Baba S and Nakamura Y (1995) Detection of K-*ras* mutations in DNAs isolated from feces of patients with colorectal tumors by mutant-allele-specific amplification (MASA) *Oncogene* **10**: 1441–1445

Hayashi N, Ito I, Yanagisawa A, Kato Y, Nakamori S, Imaoka S, Watanabe H, Ogawa M and Nakamura Y (1995) Genetic diagnosis of lymph-node metastasis in colorectal cancer. *Lancet* **345**: 1257–1259

Houlston RS, Collins A, Slack J and Morton NE (1992) Dominant genes for colorectal cancer are not rare. *Ann HumGenet* **56**: 99–103

Jones PA and Buckley JD (1990) The role of DNA methylation in cancer. *Adv Cancer Res* **54**: 1–23

Kern SE, Fearon ER, Tersmette KWF, Enterline JP, Leppert M, Nakamura Y, White R, Vogelstein B and Hamilton SR (1989) Allelic loss in colorectal carcinoma. *J Am Med Assoc* **261**: 3099–3103

Kwok S and Higuchi R (1989) Avoiding false positives with PCR. *Nature* **339**: 237–238

Laird PW, Jacksongrusby L, Fazeli A, Dickson SL, Jung WE, Li E, Weinberg RA and Jaenisch R (1995) Suppression of intestinal neoplasia by DNA hypomethylation. *Cell* **81**: 197–205

Laurent-Puig P, Olschwang S, Delattre O, Remvikos Y, Asselain B, Melot T, Validire P, Muleris M, Girodet J, Salmon RJ and Thomas G (1992) Survival and acquired genetic alterations in colorectal cancer. *Gastroenterology* **102**: 1136–1141

Levi S, Urbano-Ispizua A, Gill R, Thomas DM, Gilbertson J, Foster C and Marshall CJ (1991) Multiple K-*ras* codon 12 mutations in cholangiocarcinomas demonstrated with a sensitive polymerase chain reaction technique. *Cancer Res* **51**: 3497–3502

Lewis CM, Neuhausen DD, Black FJ, Swensen J, Burt RW, Cannon-Albright LA and Skolnick MH (1996) Genetic heterogeneity and unmapped genes for colorectal cancer. *Cancer Res* **56**: 1382–1388

Macklin MT (1960) Inheritance of cancer of the stomach and large intestine in man. *J Natl Cancer Inst* **24**: 551–571

Magewu AN and Jones PA (1994) Ubiquitous and tenacious methylation of the CpG site in codon-248 of the *p53* gene may explain its frequent appearance as a mutational hot-spot in human cancer. *Mol Cell Biol* **14**: 4225–4232

McLellan EA, Owen RA, Stepniewska KA, Sheffield JP and Lemoine NR (1993) High frequency of K-*ras* mutations in sporadic colorectal adenomas. *Gut* **34**: 392–396

Moerkerk P, Arends JW, Van Driel M, De Bruïne A, De Goeij A and Ten Kate J (1994) Type and number of Ki-*ras* point mutations relate to stage of human colorectal cancer. *Cancer Res* **54**: 3376–3378

Morrin M, Kelly M, Barrett N and Delaney P (1994) Mutations of Ki-*ras* and p53 genes in colorectal cancer and their prognostic significance. *Gut* **35**: 1627–1631

Newton CR, Kalsheker N, Graham A, Powell S, Gammack A, Riley J and Markham AF (1988) Diagnosis of $\alpha_1$-antitrypsin deficiency by enzymatic amplification of human genomic DNA and direct sequencing of polymerase chain reaction products. *Nucleic Acid Res* **16**: 8233–8243

Newton CR, Graham A, Heptinstall LE, Powell SJ, Summers C, Kalsheker N, Smith JC and Markham AF (1989) Analysis of any point mutation in DNA. The amplification refractory mutation system (ARMS). *Nucleic Acid Res* **17**: 2503–2516

Offerhaus GJA, De Feyter EP, Cornelisse CJ, Tersmette KWF, Floyd J, Kern SE, Vogelstein B and Hamilton SR (1992) The relationship of DNA aneuploidy to molecular genetic alterations in colorectal carcinoma. *Gastroenterology* **102**: 1612–1619

Oudejans JJ, Slebos RJC, Zoetmulder FAN, Mooi WJ and Rodenhuis S (1991) Differential activation of *ras* genes by point mutation in human colon cancer with metastases to either lung or liver. *Int J Cancer* **49**: 875–879

Peltomaki P, Aaltonen LA, Sistonen P, Pylkkanene L, Mecklin J-P, Jarvinen H, Green JS, Jass JR, Weber JL, Leach JL, Leach FS, Petersen GM, Hamilton SR, De La Chapelle A and Vogelstein B (1993) Genetic mapping of a locus predisposing to human colorectal cancer. *Science* **260**: 810–812

Petersen GM (1995) Genetic epidemiology of colorectal cancer. *Eur J Cancer* **31A**: 1047–1050

Ranaldi R, Giocchini AM, Manzin A, Clementi M, Stefania P and Bearzi I (1995) Adenoma–carcinoma sequence of colorectum. *Diagn Mol Pathol* **4**:198–202

Redston MS and Kern SE (1994) Klenow co-sequencing: a method for eliminating 'stops'. *Biotechniques* **17**: 286–288

Ries LAG, Miller BA, Hankey BF, Kosary CL, Harras A and Edwards BK (1994) *Surveillance, Epidemiology and End Results (SEER) Cancer Statistics Review, 1973–1991: Tables and Graphs, National Cancer Institute*. NIH publication no. 94–2789. Bethesda, USA

© Cancer Research Campaign 1998

Riley J, Jenner D, Smith JC and Markham AF (1989) Rapid determination of DNA concentration in multiple samples. *Nucleic Acid Res* **17**: 8383

Riordan JR, Rommens JM, Kerem B, Alon N, Rozmahel R, Grzelczak Z, Zielenski J, Lok S, Plavsic N, Chou J, Drumm MJ, Ianuzzi MC, Collins FS and Tsui L (1989) Identification of the cystic fibrosis gene: cloning and characterization of complementary DNA. *Science* **245**: 1066–1073

Sambrook J, Fritsch EF and Maniatis T (1989) *Molecular Cloning: A Laboratory Manual*. 2nd edn. Cold Spring Harbor Laboratory Press: Cold Spring Harbor, NY

Sharrard RM, Royds JA, Rogers S and Shorthouse AJ (1992) Patterns of methylation of the c-*myc* gene in human colorectal cancer progression. *Br J Cancer* **65**: 667–672

Sidransky D, Tokino T, Hamilton SR, Kinzler KK, Levin B, Frost P and Vogelstein B (1992) Identification of *ras* oncogene mutations in the stool of patients with curable colorectal tumors. *Science* **256**: 102–105

Span M, Moerker PTM, De Goeij AFPM and Arends JW (1996) A detailed analysis of K-*ras* mutations in relation to tumor progression and survival in colorectal cancer patients. *Int J Cancer* **69**: 241–245

Stork P, Loda M, Bosari S, Wiley B, Poppenhusen K and Wolfe H (1991) Detection of K-*ras* mutations in pancreatic and hepatic neoplasms by non-isotopic mismatched polymerase chain reaction. *Oncogene* **6**: 857–862

Tanaka M, Omura K, Watanabe Y, Oda Y and Nakanishi I (1994) Prognostic factors of colorectal cancer: K-*ras* mutation, overexpression of the p53 protein, and cell proliferative activity. *J Surg Oncol* **57**: 57–64

Urosevic N, Krtolica K, Scaro-Milic A, Knezevic-Usai S and Dujic A (1993) Prevalence of G-to-T transversions among K-*ras* oncogene mutations in human colorectal cancer in Yugoslavia. *Int J Cancer* **54**: 249–254

Villa E, Dugani A, Rebecchi AM, Vignoli A, Grottola A, Buttafoco P, Losi L, Perini M, Trande P, Merighi A, Lerose R and Manenti F (1996) Identification of subjects at risk for colorectal carcinoma through a test based on K-*ras* determination in the stool. *Gastroenterology* **110**: 1346–1353

Vogelstein B, Fearon ER, Hamilton SR, Kern SE, Preisinger AC, Leppert M, Nakamura Y, White R, Smits AMM and Bos JL (1988) Genetic alterations during colorectal tumor development. *N Engl J Med* **319**: 525–532

Vogelstein B, Fearon ER, Kern SE, Hamilton SR, Preisinger AC, Nakamura Y and White R (1989) Allelotype of colorectal carcinomas. *Science* **244**: 207–211

Woolf CM (1958) A genetic study of carcinoma of the large intestine. *Am J Hum Genet* **10**: 42–47

Yamada H, Yoshida T, Sakamoto H, Terada M and Sugimura T (1986) Establishment of a human pancreatic adenocarcinoma cell line (PSN-1) with amplifications of both c-*myc* and activated c-Ki-*ras* by a point mutation. *Biochem Biophys Res Commun* **140**: 167–173

Young G and Levin B (1996) Report of UICC colorectal cancer screening workshop. *Int J Cancer* **65**: 567–568

Zhao LP and Le Marchand L (1992) An analytical method for assessing patterns of familial aggregation in case–control studies. *Genet Epidemiol* **9**: 141–154

© Cancer Research Campaign 1998